# 4-Amino-3-(4-chloro­phen­yl)-1*H*-1,2,4-triazole-5(4*H*)-thione

**DOI:** 10.1107/S1600536812000785

**Published:** 2012-01-18

**Authors:** Sampath Natarajan, Rita Mathews

**Affiliations:** a Department of Advanced Technology Fusion, Konkuk University, 1 Hwayang-dong, Gwangjin-gu, Seoul 143 701, Republic of Korea

## Abstract

In the title compound, C_8_H_7_ClN_4_S, the benzene ring is statistically disordered over two conformations rotated about the Cl—C⋯C—C axis, which subtend dihedral angles of 24.7 (3) and 9.9 (2) ° with respect to the triazole ring. An intra­molecular C—H⋯N close contact occurs. In the crystal, N—H⋯N and N—H⋯S hydrogen bonds link the mol­ecules into (001) sheets: *R*_2_^2^(8) and *R*_2_^2^(10) graph-set motifs result. Weak C—H⋯N hydrogen bonds and aromatic π–π stacking inter­actions [shortest centroid–centroid separation = 3.681 (7) Å] complete the structure.

## Related literature

For a related structure and background references, see: Natarajan & Mathews (2011 ▶). For a related structure, see: Ambalavanan *et al.* (2003 ▶).
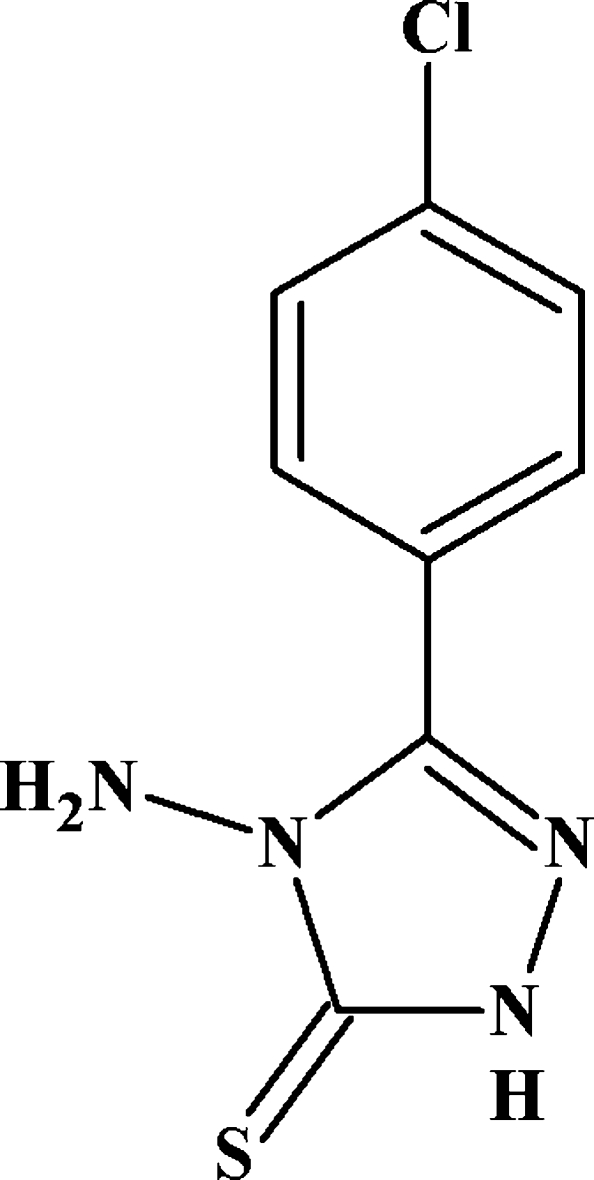


## Experimental

### 

#### Crystal data

C_8_H_7_ClN_4_S
*M* *_r_* = 226.69
Triclinic, 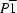

*a* = 6.0765 (9) Å
*b* = 8.0268 (7) Å
*c* = 10.9873 (16) Å
α = 72.501 (10)°
β = 87.597 (10)°
γ = 67.88 (2)°
*V* = 471.94 (12) Å^3^
*Z* = 2
Cu *K*α radiation
μ = 5.35 mm^−1^
*T* = 293 K
0.24 × 0.18 × 0.12 mm

#### Data collection

Enraf–Nonius CAD-4 diffractometer
Absorption correction: part of the refinement model (Δ*F*) (*SHELXA*; Sheldrick, 2008) ▶ *T*_min_ = 0.146, *T*_max_ = 0.618
1912 measured reflections
1818 independent reflections
1670 reflections with *I* > 2σ(*I*)
*R*_int_ = 0.054
3 standard reflections every 60 min intensity decay: none

#### Refinement

*R*[*F* ^2^ > 2σ(*F* ^2^)] = 0.080
*wR*(*F* ^2^) = 0.236
*S* = 1.09
1818 reflections
173 parameters
H atoms treated by a mixture of independent and constrained refinement
Δρ_max_ = 0.62 e Å^−3^
Δρ_min_ = −0.51 e Å^−3^

### 

Data collection: *CAD-4 EXPRESS* (Enraf–Nonius, 1994 ▶); cell refinement: *CAD-4 EXPRESS*; data reduction: *XCAD4* (Harms, 1996 ▶); program(s) used to solve structure: *SHELXS97* (Sheldrick, 2008 ▶); program(s) used to refine structure: *SHELXL97* (Sheldrick, 2008 ▶); molecular graphics: *ORTEP-3* (Farrugia, 1997 ▶) and *PLATON* (Spek, 2009 ▶); software used to prepare material for publication: *SHELXL97* and *PLATON*.

## Supplementary Material

Crystal structure: contains datablock(s) I, global. DOI: 10.1107/S1600536812000785/hb6583sup1.cif

Structure factors: contains datablock(s) I. DOI: 10.1107/S1600536812000785/hb6583Isup2.hkl

Supplementary material file. DOI: 10.1107/S1600536812000785/hb6583Isup3.cml

Additional supplementary materials:  crystallographic information; 3D view; checkCIF report

## Figures and Tables

**Table 1:** Hydrogen-bond geometry (Å, °)

*D*—H⋯*A*	*D*—H	H⋯*A*	*D*⋯*A*	*D*—H⋯*A*
C4*B*—H4*B*⋯N4	0.93	2.32	2.98	128
N3—H3⋯S1^i^	0.86	2.55	3.332 (3)	152
C5*B*—H5*B*⋯N4^ii^	0.93	2.72	3.582 (8)	155
C8*A*—H8*A*⋯N4^iii^	0.93	2.53	3.416 (9)	158
C7*B*—H7*B*⋯N2^iv^	0.93	2.64	3.541 (10)	162
N4—H4*C*⋯S1^v^	0.91 (6)	2.70 (6)	3.552 (4)	155 (5)
N4—H4*D*⋯N2^vi^	0.96 (7)	2.42 (7)	3.349 (5)	164 (5)

Symmetry codes: (i) 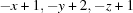; (ii) 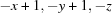; (iii) 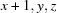; (iv) 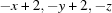; (v) 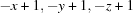; (vi) 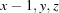.

**Fig. 1.**
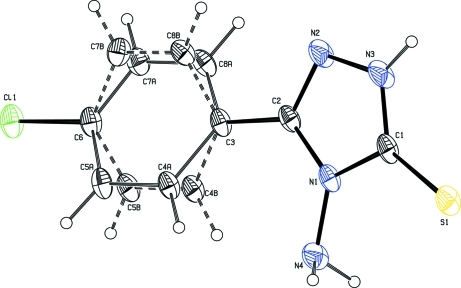
ORTEP diagram of the title molecule with displacement ellipsoid drawn at 30% probability level.

**Fig. 2.**
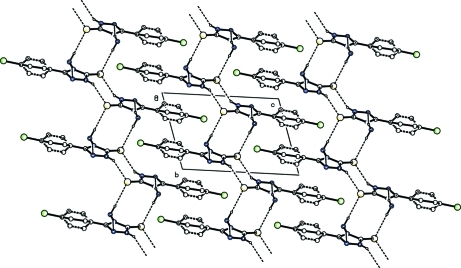
A unit cell packing of the crystal structure of the title compound viewed down a axis. Dashed lines are indicating the hydrogen bonds between the molecules.

## supplementary crystallographic information

### Comment

As part of our onging studies of 1,2,4-triazole derivatives (Natarajan & Mathews, 2011), we now describe the title compound.

The title molecule (Fig.1) contains the rings 1,2,4-triazole and 4-chlorophenyl. All the bond lengths and bond angles are well agreed with previously reported structure (Ambalavanan *et al.*, 2003; Natarajan and Mathews, 2011). The phenyl ring substituted on the atom C2 shows rotational disorder by the atoms of C4, C5, C7 & C8. The rotational disorder of phenyl ring results in two phenyl ring orientations A (C3/C4A/C5A/C6/C7A/C8A) and B (C3/C4B/C5B/CB/C7B/C8B) with respect to 1,2,4-triazole ring. The dihedral angles of these phenyl rings with triazole moiety are 24.7 (3) and 9.9 (2)°, respectively for phenyl rings A and B.

The packing diagram of the title compound viewed down *a* axis is shown in Fig. 2. The crystal structure is stabilized by the intra and intermolecular hydrogen bonds namely N—H···N, C—H···N and N—H···S. One of them (N—H···S) is involved in self-complementary interactions of triazole rings and forms *R*_2_^2^(8) and *R*_2_^2^(10) types graph set motifs. The motif, *R*_2_^2^(10) is formed by the dimer interactions between the symmetry related 1,2,4-triazole-3-thione moieties and it is connected by the motif *R*_2_^2^(8) along the *b* axis in the unit cell packing. In addition, three π···π weak interactions {*Cg*1···*Cg*1= 3.681 (7) Å, *Cg*1···*Cg*2= 3.691 (7)Å & *Cg*2···*Cg*2 = 3.701 (7)Å (2 - *x*, 1 - *y*, -*z*); *Cg* is the centroid of the rings; *Cg*1= C3/C4A/C5A/C6/C7A/C8A and *Cg*2= C3/C4B/C5B/C6/C7B/C8B} are also helping to consolidate the molecules in crystal packing. The detailed geometry of the non-bonded interactions is presented in Table 1.

### Experimental

A mixture of β-4-chlorophenyldithiocarbazinate potassium salt (0.1 mol) and hydrazine hydrate (0.25 mol) was heated on an oil-bath at 150° C for 5 h (until evolution of H2S gas in the reaction). The reaction mixture was then cooled and poured into the cold water and then acidified with conc. HCl. The filtered product was washed extensively with cold water and recrystallized using ethanol to yield yellow needles.

### Refinement

The primary amine H atoms were derived from the Fourier map and the remaining H atoms were positioned geometrically and refined using a riding model with C—H = 0.93 Å for aromatic Hs and for N—H = 0.86 Å. The *U*_iso_ values were constrained to be 1.2Ueq of the carrier atom for the aromatic C—H and N—H hydrogen atoms.

### Crystal data

**Table:**

C_8_H_7_ClN_4_S	*Z* = 2
*M**_r_* = 226.69	*F*(000) = 232
Triclinic, *P*1	*D*_x_ = 1.595 Mg m^−^^3^
*a* = 6.0765 (9) Å	Cu *K*α radiation, λ = 1.54184 Å
*b* = 8.0268 (7) Å	Cell parameters from 25 reflections
*c* = 10.9873 (16) Å	θ = 1–60°
α = 72.501 (10)°	µ = 5.35 mm^−^^1^
β = 87.597 (10)°	*T* = 293 K
γ = 67.88 (2)°	Needle, yellow
*V* = 471.94 (12) Å^3^	0.24 × 0.18 × 0.12 mm

### Data collection

**Table:**

Enraf–Nonius CAD-4 diffractometer	1670 reflections with *I* > 2σ(*I*)
Radiation source: fine-focus sealed tube	*R*_int_ = 0.054
graphite	θ_max_ = 72.5°, θ_min_ = 4.2°
ω scans	*h* = −6→7
Absorption correction: part of the refinement model (Δ*F*) (SHELXA; Sheldrick, 2008)	*k* = −9→9
*T*_min_ = 0.146, *T*_max_ = 0.618	*l* = −9→13
1912 measured reflections	3 standard reflections every 60 min
1818 independent reflections	intensity decay: none

### Refinement

**Table:**

Refinement on *F*^2^	Secondary atom site location: difference Fourier map
Least-squares matrix: full	Hydrogen site location: inferred from neighbouring sites
*R*[*F*^2^ > 2σ(*F*^2^)] = 0.080	H atoms treated by a mixture of independent and constrained refinement
*wR*(*F*^2^) = 0.236	*w* = 1/[σ^2^(*F*_o_^2^) + (0.1913*P*)^2^ + 0.1469*P*] where *P* = (*F*_o_^2^ + 2*F*_c_^2^)/3
*S* = 1.09	(Δ/σ)_max_ < 0.001
1818 reflections	Δρ_max_ = 0.62 e Å^−^^3^
173 parameters	Δρ_min_ = −0.51 e Å^−^^3^
0 restraints	Extinction correction: *SHELXL97* (Sheldrick, 2008), Fc^*^=kFc[1+0.001xFc^2^λ^3^/sin(2θ)]^-1/4^
Primary atom site location: structure-invariant direct methods	Extinction coefficient: 0.031 (8)

### Special details

**Table:**

Geometry. All e.s.d.'s (except the e.s.d. in the dihedral angle between two l.s. planes) are estimated using the full covariance matrix. The cell e.s.d.'s are taken into account individually in the estimation of e.s.d.'s in distances, angles and torsion angles; correlations between e.s.d.'s in cell parameters are only used when they are defined by crystal symmetry. An approximate (isotropic) treatment of cell e.s.d.'s is used for estimating e.s.d.'s involving l.s. planes.
Refinement. Refinement of *F*^2^ against ALL reflections. The weighted *R*-factor *wR* and goodness of fit *S* are based on *F*^2^, conventional *R*-factors *R* are based on *F*, with *F* set to zero for negative *F*^2^. The threshold expression of *F*^2^ > σ(*F*^2^) is used only for calculating *R*-factors(gt) *etc*. and is not relevant to the choice of reflections for refinement. *R*-factors based on *F*^2^ are statistically about twice as large as those based on *F*, and *R*- factors based on ALL data will be even larger.

### Fractional atomic coordinates and isotropic or equivalent isotropic displacement parameters (Å^2^)

**Table:**

	*x*	*y*	*z*	*U*_iso_*/*U*_eq_	Occ. (<1)
Cl1	0.91511 (17)	0.68117 (13)	−0.30339 (8)	0.0585 (5)	
S1	0.31850 (16)	0.79307 (13)	0.50686 (8)	0.0534 (5)	
N1	0.5022 (5)	0.7466 (4)	0.2844 (2)	0.0404 (7)	
N2	0.7683 (6)	0.8756 (5)	0.2550 (3)	0.0569 (9)	
N3	0.6640 (6)	0.8820 (5)	0.3677 (3)	0.0596 (9)	
H3	0.7032	0.9292	0.4196	0.072*	
N4	0.3603 (7)	0.6528 (5)	0.2662 (3)	0.0541 (8)	
C1	0.4998 (6)	0.8091 (5)	0.3885 (3)	0.0453 (8)	
C2	0.6627 (5)	0.7938 (4)	0.2044 (3)	0.0407 (7)	
C3	0.7171 (5)	0.7637 (4)	0.0791 (3)	0.0395 (7)	
C4A	0.5509 (19)	0.7474 (19)	0.0004 (9)	0.0404 (19)	0.50 (2)
H4A	0.4041	0.7494	0.0291	0.048*	0.50 (2)
C5A	0.6105 (19)	0.7284 (18)	−0.1214 (9)	0.048 (2)	0.50 (2)
H5A	0.5020	0.7227	−0.1757	0.057*	0.50 (2)
C4B	0.631 (2)	0.661 (2)	0.0331 (10)	0.041 (2)	0.50 (2)
H4B	0.5303	0.6072	0.0799	0.050*	0.50 (2)
C5B	0.694 (2)	0.636 (2)	−0.0856 (10)	0.047 (3)	0.50 (2)
H5B	0.6401	0.5606	−0.1156	0.056*	0.50 (2)
C6	0.8321 (6)	0.7185 (4)	−0.1578 (3)	0.0437 (8)	
C7A	0.9891 (19)	0.737 (2)	−0.0863 (8)	0.047 (2)	0.50 (2)
H7A	1.1338	0.7384	−0.1168	0.057*	0.50 (2)
C8A	0.9324 (19)	0.755 (2)	0.0335 (8)	0.047 (2)	0.50 (2)
H8A	1.0451	0.7608	0.0850	0.057*	0.50 (2)
C7B	0.9186 (19)	0.831 (2)	−0.1110 (9)	0.046 (2)	0.50 (2)
H7B	1.0154	0.8880	−0.1592	0.056*	0.50 (2)
C8B	0.8574 (18)	0.856 (2)	0.0063 (8)	0.041 (2)	0.50 (2)
H8B	0.9085	0.9321	0.0371	0.050*	0.50 (2)
H4C	0.394 (11)	0.538 (9)	0.327 (6)	0.095 (19)*	
H4D	0.198 (12)	0.724 (9)	0.277 (6)	0.096 (18)*	

### Atomic displacement parameters (Å^2^)

**Table:**

	*U*^11^	*U*^22^	*U*^33^	*U*^12^	*U*^13^	*U*^23^
Cl1	0.0650 (7)	0.0717 (7)	0.0365 (6)	−0.0151 (5)	0.0119 (4)	−0.0289 (4)
S1	0.0548 (7)	0.0735 (7)	0.0408 (6)	−0.0209 (5)	0.0126 (4)	−0.0356 (5)
N1	0.0407 (14)	0.0474 (13)	0.0337 (13)	−0.0068 (10)	0.0014 (10)	−0.0258 (11)
N2	0.0591 (18)	0.090 (2)	0.0414 (16)	−0.0351 (16)	0.0129 (13)	−0.0406 (15)
N3	0.0627 (19)	0.098 (2)	0.0446 (16)	−0.0400 (17)	0.0151 (14)	−0.0490 (17)
N4	0.0587 (19)	0.073 (2)	0.0469 (17)	−0.0299 (15)	0.0138 (14)	−0.0360 (15)
C1	0.0451 (17)	0.0584 (17)	0.0326 (15)	−0.0081 (14)	0.0023 (12)	−0.0295 (13)
C2	0.0359 (15)	0.0500 (16)	0.0358 (15)	−0.0072 (12)	−0.0010 (11)	−0.0238 (12)
C3	0.0373 (15)	0.0435 (14)	0.0330 (14)	−0.0024 (11)	0.0001 (11)	−0.0213 (11)
C4A	0.033 (4)	0.049 (5)	0.035 (4)	−0.003 (4)	0.001 (3)	−0.023 (3)
C5A	0.050 (4)	0.051 (5)	0.033 (4)	−0.005 (4)	−0.003 (3)	−0.018 (4)
C4B	0.041 (4)	0.053 (6)	0.034 (4)	−0.012 (4)	0.008 (3)	−0.027 (4)
C5B	0.052 (5)	0.057 (6)	0.038 (4)	−0.016 (5)	0.004 (4)	−0.030 (4)
C6	0.0419 (16)	0.0468 (15)	0.0338 (16)	−0.0026 (13)	0.0028 (12)	−0.0188 (12)
C7A	0.047 (4)	0.054 (6)	0.040 (4)	−0.014 (4)	0.003 (3)	−0.020 (4)
C8A	0.045 (4)	0.055 (6)	0.045 (4)	−0.012 (4)	−0.004 (3)	−0.028 (4)
C7B	0.045 (4)	0.048 (6)	0.040 (4)	−0.011 (4)	0.010 (3)	−0.016 (4)
C8B	0.041 (4)	0.048 (6)	0.042 (4)	−0.012 (4)	0.006 (3)	−0.030 (4)

### Geometric parameters (Å, °)

**Table:**

Cl1—C6	1.735 (3)	C4A—C5A	1.410 (9)
S1—C1	1.675 (3)	C4A—H4A	0.9300
N1—C2	1.368 (4)	C5A—C6	1.368 (9)
N1—C1	1.378 (4)	C5A—H5A	0.9300
N1—N4	1.394 (4)	C4B—C5B	1.395 (9)
N2—C2	1.311 (4)	C4B—H4B	0.9300
N2—N3	1.373 (4)	C5B—C6	1.346 (9)
N3—C1	1.315 (5)	C5B—H5B	0.9300
N3—H3	0.8572	C6—C7A	1.339 (9)
N4—H4C	0.91 (6)	C6—C7B	1.421 (10)
N4—H4D	0.96 (7)	C7A—C8A	1.381 (10)
C2—C3	1.471 (4)	C7A—H7A	0.9300
C3—C4B	1.344 (8)	C8A—H8A	0.9300
C3—C8A	1.365 (9)	C7B—C8B	1.377 (10)
C3—C8B	1.405 (9)	C7B—H7B	0.9300
C3—C4A	1.423 (8)	C8B—H8B	0.9300
			
C2—N1—C1	108.7 (3)	C6—C5A—H5A	120.8
C2—N1—N4	127.1 (3)	C4A—C5A—H5A	120.8
C1—N1—N4	124.3 (3)	C3—C4B—C5B	119.3 (7)
C2—N2—N3	104.2 (3)	C3—C4B—H4B	120.4
C1—N3—N2	114.0 (3)	C5B—C4B—H4B	120.4
C1—N3—H3	123.3	C6—C5B—C4B	121.7 (6)
N2—N3—H3	122.7	C6—C5B—H5B	119.1
N1—N4—H4C	113 (4)	C4B—C5B—H5B	119.1
N1—N4—H4D	109 (4)	C7A—C6—C5B	112.0 (5)
H4C—N4—H4D	104 (5)	C7A—C6—C5A	123.0 (5)
N3—C1—N1	103.3 (3)	C5B—C6—C5A	30.8 (4)
N3—C1—S1	131.5 (3)	C7A—C6—C7B	28.6 (3)
N1—C1—S1	125.2 (3)	C5B—C6—C7B	119.2 (5)
N2—C2—N1	109.8 (3)	C5A—C6—C7B	113.4 (5)
N2—C2—C3	122.3 (3)	C7A—C6—Cl1	118.0 (4)
N1—C2—C3	127.9 (3)	C5B—C6—Cl1	121.1 (4)
C4B—C3—C8A	110.6 (5)	C5A—C6—Cl1	119.0 (4)
C4B—C3—C8B	121.1 (5)	C7B—C6—Cl1	119.7 (4)
C8A—C3—C8B	30.7 (3)	C6—C7A—C8A	118.8 (7)
C4B—C3—C4A	28.1 (3)	C6—C7A—H7A	120.6
C8A—C3—C4A	117.7 (5)	C8A—C7A—H7A	120.6
C8B—C3—C4A	111.9 (5)	C3—C8A—C7A	122.4 (6)
C4B—C3—C2	122.7 (4)	C3—C8A—H8A	118.8
C8A—C3—C2	119.7 (4)	C7A—C8A—H8A	118.8
C8B—C3—C2	116.1 (4)	C8B—C7B—C6	119.3 (7)
C4A—C3—C2	122.6 (4)	C8B—C7B—H7B	120.3
C5A—C4A—C3	119.5 (6)	C6—C7B—H7B	120.3
C5A—C4A—H4A	120.2	C7B—C8B—C3	119.3 (6)
C3—C4A—H4A	120.2	C7B—C8B—H8B	120.4
C6—C5A—C4A	118.3 (6)	C3—C8B—H8B	120.4
			
C2—N2—N3—C1	−0.2 (5)	C2—C3—C4B—C5B	−178.8 (6)
N2—N3—C1—N1	1.7 (4)	C3—C4B—C5B—C6	−2.7 (14)
N2—N3—C1—S1	−177.0 (3)	C4B—C5B—C6—C7A	31.8 (11)
C2—N1—C1—N3	−2.5 (3)	C4B—C5B—C6—C5A	−86.1 (12)
N4—N1—C1—N3	178.0 (3)	C4B—C5B—C6—C7B	1.1 (11)
C2—N1—C1—S1	176.3 (2)	C4B—C5B—C6—Cl1	178.4 (7)
N4—N1—C1—S1	−3.2 (4)	C4A—C5A—C6—C7A	−4.1 (11)
N3—N2—C2—N1	−1.4 (4)	C4A—C5A—C6—C5B	73.4 (10)
N3—N2—C2—C3	178.1 (3)	C4A—C5A—C6—C7B	−34.8 (9)
C1—N1—C2—N2	2.5 (4)	C4A—C5A—C6—Cl1	176.3 (5)
N4—N1—C2—N2	−178.0 (3)	C5B—C6—C7A—C8A	−28.2 (10)
C1—N1—C2—C3	−176.9 (3)	C5A—C6—C7A—C8A	4.5 (11)
N4—N1—C2—C3	2.5 (5)	C7B—C6—C7A—C8A	82.8 (12)
N2—C2—C3—C4B	172.1 (9)	Cl1—C6—C7A—C8A	−175.9 (6)
N1—C2—C3—C4B	−8.5 (9)	C4B—C3—C8A—C7A	32.1 (10)
N2—C2—C3—C8A	24.1 (9)	C8B—C3—C8A—C7A	−84.4 (11)
N1—C2—C3—C8A	−156.5 (8)	C4A—C3—C8A—C7A	2.2 (11)
N2—C2—C3—C8B	−10.5 (7)	C2—C3—C8A—C7A	−176.4 (7)
N1—C2—C3—C8B	168.9 (7)	C6—C7A—C8A—C3	−3.5 (13)
N2—C2—C3—C4A	−154.4 (7)	C7A—C6—C7B—C8B	−83.4 (12)
N1—C2—C3—C4A	25.0 (8)	C5B—C6—C7B—C8B	−0.8 (10)
C4B—C3—C4A—C5A	−83.7 (11)	C5A—C6—C7B—C8B	33.1 (9)
C8A—C3—C4A—C5A	−1.8 (10)	Cl1—C6—C7B—C8B	−178.1 (5)
C8B—C3—C4A—C5A	31.6 (9)	C6—C7B—C8B—C3	2.0 (11)
C2—C3—C4A—C5A	176.8 (6)	C4B—C3—C8B—C7B	−3.6 (10)
C3—C4A—C5A—C6	2.6 (11)	C8A—C3—C8B—C7B	74.2 (9)
C8A—C3—C4B—C5B	−28.3 (10)	C4A—C3—C8B—C7B	−33.5 (9)
C8B—C3—C4B—C5B	3.9 (11)	C2—C3—C8B—C7B	178.9 (6)
C4A—C3—C4B—C5B	82.2 (13)		

### Hydrogen-bond geometry (Å, °)

**Table:**

*D*—H···*A*	*D*—H	H···*A*	*D*···*A*	*D*—H···*A*
C4B—H4B···N4	0.93	2.32	2.98	128
N3—H3···S1^i^	0.86	2.55	3.332 (3)	152
C5B—H5B···N4^ii^	0.93	2.72	3.582 (8)	155
C8A—H8A···N4^iii^	0.93	2.53	3.416 (9)	158
C7B—H7B···N2^iv^	0.93	2.64	3.541 (10)	162
N4—H4C···S1^v^	0.91 (6)	2.70 (6)	3.552 (4)	155 (5)
N4—H4D···N2^vi^	0.96 (7)	2.42 (7)	3.349 (5)	164 (5)

Symmetry codes: (i) −*x*+1, −*y*+2, −*z*+1; (ii) −*x*+1, −*y*+1, −*z*; (iii) *x*+1, *y*, *z*; (iv) −*x*+2, −*y*+2, −*z*; (v) −*x*+1, −*y*+1, −*z*+1; (vi) *x*−1, *y*, *z*.

## References

[bb1] Ambalavanan, P., Palani, K., Ponnuswamy, M. N., Thirumuruhan, R. A., Yathirajan, S. H., Prabhuswamy, B., Raju, C. R., Nagaraja, P. & Mohana, K. N. (2003). *Mol. Cryst. Liq. Cryst.* **393**, 67–73.

[bb2] Enraf–Nonius (1994). *CAD-4 EXPRESS* Enraf–Nonius, Delft, The Netherlands.

[bb3] Farrugia, L. J. (1997). *J. Appl. Cryst.* **30**, 565.

[bb4] Harms, K. (1996). *XCAD4* University of Marburg, Germany.

[bb5] Natarajan, S. & Mathews, R. (2011). *Acta Cryst.* E**67**, o2828. 10.1107/S1600536811039833 PMC3247567 22219872

[bb6] Sheldrick, G. M. (2008). *Acta Cryst.* A**64**, 112–122. 10.1107/S0108767307043930 18156677

[bb7] Spek, A. L. (2009). *Acta Cryst.* D**65**, 148–155. 10.1107/S090744490804362X PMC2631630 19171970

